# Performance Improvement Effect of Asphalt Binder Using Pyrolysis Carbon Black

**DOI:** 10.3390/ma15124158

**Published:** 2022-06-11

**Authors:** Kwanho Lee, Seongkyum Kim

**Affiliations:** Department of Civil Engineering, Kongju National University, Cheonan Dae Ro 1223-24, Seobuk-Gu, Cheonan-si 31080, Korea; tjdrua0614@kongju.ac.kr

**Keywords:** asphalt binder, pyrolysis carbon black, dynamic shear modulus, fatigue, creep stiffness

## Abstract

The generation of waste tires is rapidly increasing. Waste tire pyrolysis is an alternative to waste tire recycling. The main substances extracted in the waste tire pyrolysis method include oil, carbon black, and iron. In this study, carbon black from the pyrolysis of waste tires was used to modify and improve the permanent deformation properties of the asphalt binder, and 0%, 5%, 10%, 15%, and 20% of pyrolysis carbon black by weight were mixed in raw asphalt, which is called AP-3 and AP-5. Laboratory tests, such as the softening point test, flash point test, rotational viscometer test, dynamic shear rheometer test, and bending beam rheometer test, were carried out. The use of pyrolysis carbon black increased the softening point and rotational viscosity at 135 °C. When using 15% PCB for AP-3 and 10% PCB for AP-5, the performance improvement effect of the resistance to permanent deformation was significant. The use of pyrolysis carbon black decreased fatigue at room temperature and improved the resistance of low-temperature cracking up to −12 °C but gave poor results at −18 °C.

## 1. Introduction

Among the solid wastes in Korea, waste tires are bulky and prolific in number, which causes many problems with their disposal. Due to the development of the economy, the increase in the number of vehicles owned by individuals and institutions has increased rapidly. Table 1 shows the number of waste tires generated by year [1]. About 75% of waste tires are generated when replacing tires, and about 25% of waste tires are generated when a car is scrapped in Korea.

The waste tires generated in this way cause social problems that threaten our living environment. Since waste tire disposal has emerged as a social and environmental issue, the government implemented the “Responsible Recycling System for Producers” in 2003. This indicates the amount of waste to be recycled by the producer, and the producer must pay a recycling fee equivalent to 30% of the recycling cost if the target amount is not achieved. According to the Waste Management Act in March 1991 and the Enactment of the Act on Resource Saving and Recycling Promotion in December 1992, tires were made to be collected and disposed of by manufacturers/importers of products and packaging that are easily recycled. According to the regulations, tires are classified as an item subject to deposit payment that returns the deposit, and the regulations stipulate that tire manufacturers/importers must fulfill their obligations to collect/dispose of waste tires. However, since it is impossible for tire manufacturers/importers to only collect/treat their own products, waste tire collection/treatment has been handled by the Korea Tire Industry Association for joint recovery/treatment since June 1991. The Ministry of the Environment calculated and reported that the tire recycling obligation in 2004 was 193,580 tons; 71.4% of the amount generated and about 84.2% of the total recycled materials were used as heat energy. The use of heat energy from waste tires is mainly used in cement manufacturing plants. The powder processing method is reported to be the most promising method for dealing with waste tires when considering the payback period and economic feasibility [2].

The rubber asphalt construction method, which uses up to 2 mm of rubber powder by crushing waste tires and mixing them with an asphalt binder at a temperature of about 200 °C, uses wet technology and dry technology. It has been reported that rubber asphalt mixtures are more effective in preventing long-term aging due to factors such as high viscosity, low stiffness at low temperatures, high reflection crack resistance, and long-term aging compared to general asphalt binders. It is reported that this process helps improve the viscoelastic properties of asphalt binder, as reported by the Washington Road Bureau’s (WADOT) results of an asphalt binder performance test using carbon black in the United States [3,4,5,6,7].

In this study, the effect of improving the performance of asphalt binder was evaluated using pyrolysis carbon black, a residue generated during pyrolysis. The pyrolysis carbon black used in this study is produced at a plant using the pyrolysis process of waste tires in Busan and is completely different from existing waste tire rubber powder. The carbon black extracted by the pyrolysis process was processed into a powder form and used. To this end, a modified asphalt binder was prepared in which the amount of pyrolysis carbon black was mixed with 0%, 5%, 10%, and 15% of the amount of asphalt binder. To verify the improved performance of the modified asphalt binder with pyrolysis carbon black from waste tires, a number of tests, including a penetration test, softening point, rotational viscometer test, dynamic shear rheometer test, and bending beam rheometer test, were carried out. 

## 2. Testing Methods and Results

### 2.1. Testing Materials

**Figure 1:**
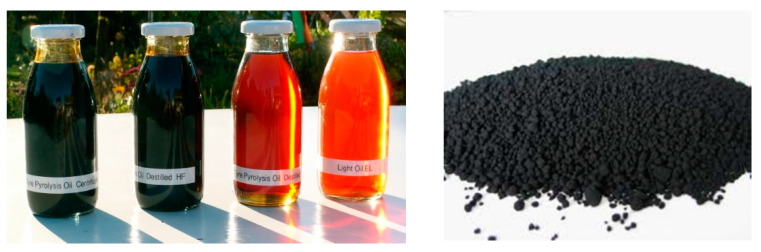
Tire pyrolysis oil and pyrolysis carbon black.

As the asphalt binder is homogeneous, the Korean Industrial Standard KS M 2201 (based on the classification of the penetration number) was used as asphalt for road pavement under the Petroleum Business Act [8]. In general, the thermal decomposition of waste tires produces 40~45% pyro-oil, 35~40% pyro-carbon, 10~15% high-quality steel, and 7~12% NC gas. Figure 1 shows the pyro-oil and pyro-carbon black. The pyrolysis carbon black used in the experiment was the pyrolysis residue of waste tires from a waste tire pyrolysis plant with a capacity of 10 tons and was provided by milling and processing the first residue. Table 2 shows the major characteristics of pyrolysis carbon black (PCB) and the other commercial carbon black in Korea. The specific gravity of the thermally decomposed carbon black was 1.000 g/cm^3^; the specific surface area was 58 m^2^/g. 

To examine the concentration of heavy metals in pyrolysis carbon black, a Pb and Ti extraction test was performed with the ASTM D5223 (1995) standard test method. After adding 0.2 to 0.5 g of the sample to a Teflon beaker, we added 20 mL of hydrofluoric acid and 20 mL of aqua regia. The extract was completely dried by heating 150 °C. 

**Figure 2:**
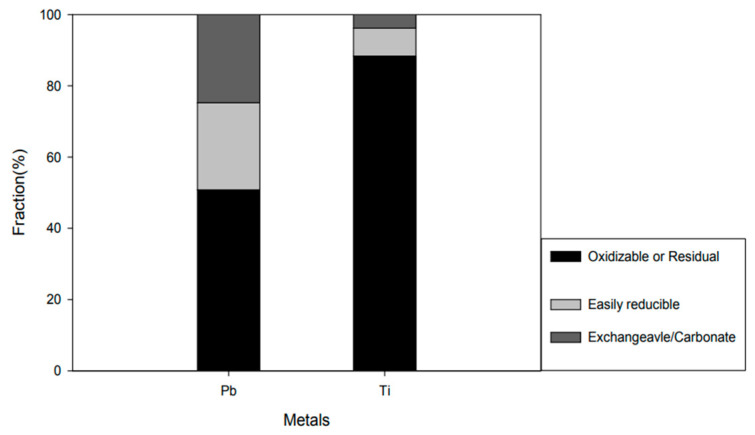
Heavy metal concentration in solids.

After drying by adding deionized water, 1 mL of nitric acid and 20 mL of deionized water were added and heated at 90 °C for 1 h. The sample was filtered under reduced pressure using a GF/C membrane filter paper with a pore size of 0.45 μm for solid–liquid separation. To identify the type of binding of heavy metals, the BCR (European Community Bureau of Reference) continuous extraction method was used. The measured concentration of heavy metal was 850~1239 for Ti and 86~490 for Pb. Figure 2 shows the heavy metal concentration in solids. 

### 2.2. Conventional Tests of Asphalt Binder

**Figure 3:**
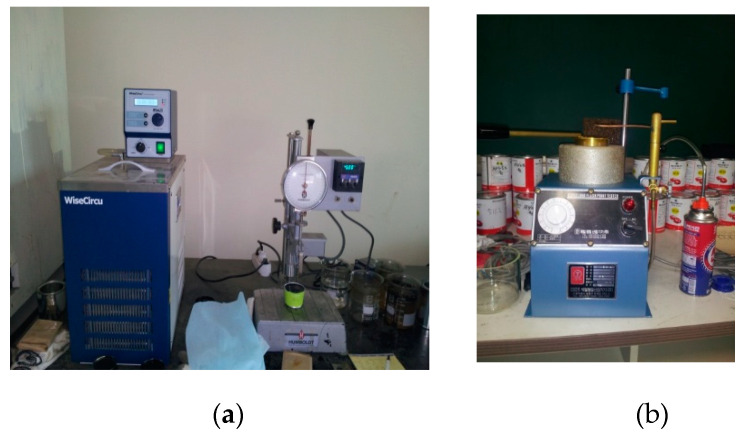
Testing setup and equipment for conventional test of asphalt binder. (**a**) Penetration test. (**b**) Flash point.

Regarding the experimental materials used in this study, an asphalt binder (AP-3 and AP-5) was used, and pyrolysis carbon black was used to improve asphalt performance. General experiments for the compatibility of the Strategic Highway Research Program (SHRP) were carried out using modified asphalt binders made of pyrolysis carbon black comprising 0%, 5%, 10%, 15%, and 20%. To verify the characteristics of the modified asphalt binder with pyrolysis carbon black, the penetration test (ASTM D5, KS M 2252), softening point test (ASTM D 648, KS M2250), and flash point test (ASTM D 92, KS M 2592) were carried out [9]. Figure 3 shows the testing setup for each test. Table 3 shows the result of the penetration test for the modified asphalt binder. The average penetration values (5 times) of AP-3 (PG 58-22) and AP-5 (PG 64-22) were 116 and 91.7, respectively. As the amount of pyrolytic carbon black used increases, the penetration value of the asphalt binder tends to decrease. Table 4 shows the measured softening point for the modified asphalt binder. The specified range of the softening point for AP-3 and AP-5 is 42 °C to 50 °C and 44 °C to 52 °C, respectively. All the test results for AP-3 and AP-5 modified asphalt binder were within the reference value range. Additionally, the measured flash points were within the specified reference value (over 260 °C).

### 2.3. SHRP Superpave PG Test and Results

#### 2.3.1. Rotational Viscometer (RV) Test

**Figure 4:**
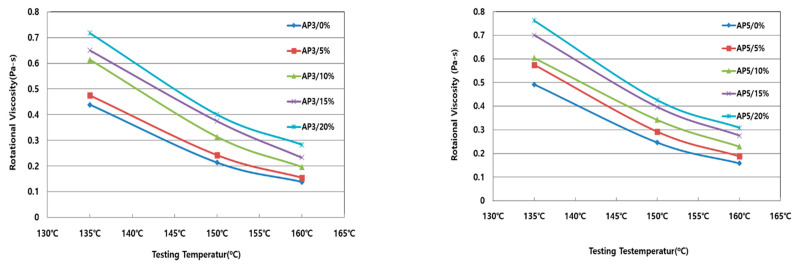
Rotational viscosity with pyrolysis carbon black.

An asphalt plant is a test that evaluates the ease of pumping asphalt, the appropriateness of mixing an aggregate and asphalt, and the appropriateness of the laying and compaction of asphalt pavement to evaluate the mixing and production of the asphalt mixture and workability during field construction. ASTM D 4402 and KS F 2392 were applied with a standard test temperature of 135 °C. Additionally, the experiment was carried out at 150 °C and 160 °C to evaluate the temperature sensitivity of the rotational viscosity according to the amount of pyrolysis carbon black. Test results for AP-3 and AP-5 modified asphalt binder are shown in Figure 4. The rotational viscosity value tended to increase with increasing pyrolysis carbon black usage. As the test temperature increased, the rotational viscosity values decreased. The experiment results were measured to 3.0 Pa-s or less at the specification of 135 °C.

#### 2.3.2. Dynamic Shear Rheometer (DSR) Test

**Figure 5:**
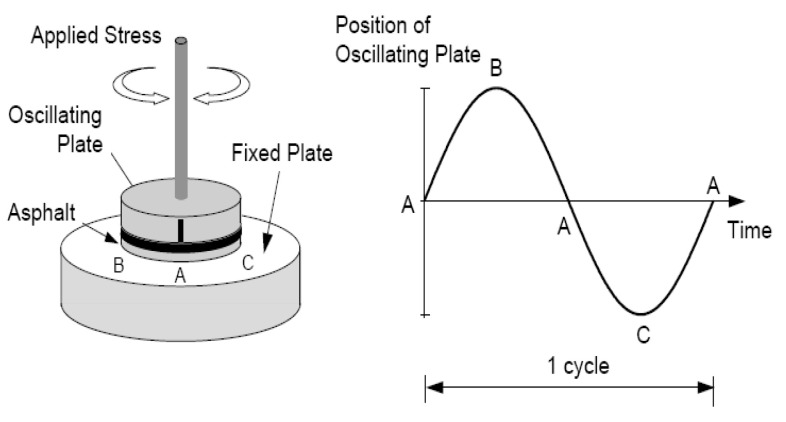
DSR test method.

Asphalt binder is a viscoelastic material with viscous and elastic properties. Viscoelastic materials are generally closely related to the temperature of the asphalt binder. When the temperature of the asphalt binder is high, it exhibits properties close to viscosity, and when the temperature of the asphalt binder is low, the properties are close to those of an elastic body. The dynamic shear flow test evaluates the plastic deformation characteristics at high temperatures and the fatigue characteristics at room temperature, and it is used to analyze the viscosity and elastic behavior characteristics of an asphalt binder in common use. Standard test methods of KS F 2393 and the SHRP Superpave PG Test were applied. The DSR test is shown in Figure 5, applying a specific gap load in the order of A-B-A-C-A [3]. The time difference in which deformation occurs when a load is applied is defined as time lag and phase lag. As the asphalt binder is a viscoelastic material, it combines a perfectly elastic body and a perfectly viscous body. The DSR test measures shear deformation by applying shear stress, which is defined as the shear modulus, G* and phase angle, δ.

The DSR test was performed at 52 °C, 58 °C, and 64 °C using the original asphalt binder, short-term aging asphalt, and long-term aging asphalt binder. The shear modulus and phase angle measured from the test are shown in Table 5 and Table 6. The measured shear modulus increased in the order of raw asphalt, short-term aged asphalt, and long-term aged asphalt, and the phase angle tended to decrease. As the amount of pyrolysis carbon black used in the same asphalt binder increased, the shear modulus increased, and the change in the phase angle showed almost similar values. As shown in Table 5 and Table 6, the shear modulus of the AP-5 modified asphalt binder was generally larger than that of the AP-3 modified asphalt binder. Table 7 shows the shear modulus and phase angle for the long-term aged asphalt binder of AP-3 and AP-5.

**Figure 6:**
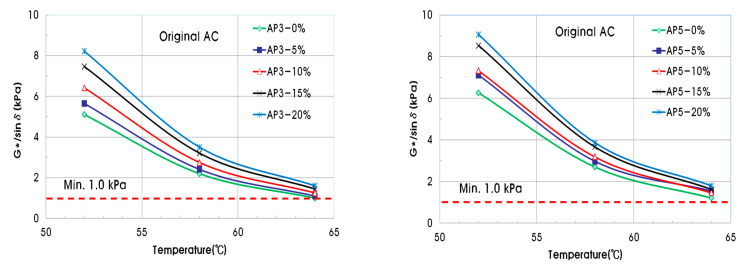
G*/sinδ of original asphalt binder for permanent deformation.

**Figure 7:**
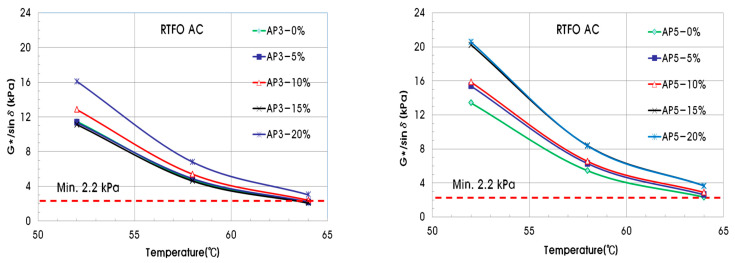
G*/sinδ of short-term aged asphalt binder for permanent deformation.

Using the shear modulus and phase angle, the G*/sinδ value for evaluating plastic deformation resistance can be determined. In the case of the original asphalt binder, the minimum standard of G*/sinδ must be 1.00 kPa or higher to be resistant to plastic deformation. The minimum value of G*/sinδ for a short-term aged asphalt binder should be 2.20 kPa or more. Figure 6 shows the G*/sinδ of the original asphalt binder with PCB, and Figure 7 shows that of the short-term aged asphalt binder. As the pyrolysis carbon black content of the original asphalt binder increases, the value of G*/sinδ also increases, and all experimental values pass the reference value of 1.00 kPa or more. In the case of the short-term aged asphalt binder, the G*/sinδ value also increased as the pyrolysis carbon black content increased. The measured values for 0% and 5% of pyrolysis carbon black did not exceed the reference value of 2.20 kPa.

Using the shear modulus and phase angle measured in the DSR test, the G*sinδ value for evaluating fatigue crack resistance can be determined. In the case of long-term aged asphalt binder, the maximum standard of G*sinδ is specified to be 5000 kPa or less. The test results (Table 8) for the different amounts of pyrolysis carbon black were 5000 kPa or less, but there was no trend of increase based on the amount of pyrolysis carbon black.

#### 2.3.3. Bending Beam Rheometer (BBR) Test

**Figure 8:**
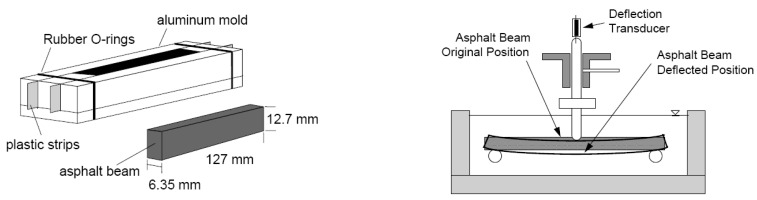
The specimen and testing setup for the BBR test.

A bending beam rheometer test was conducted to evaluate the low-temperature performance of the asphalt binder. KS F 2390 was applied as the standard test specification. Figure 8 is the standard specification of the specimen and setup used for the deflection retainer test. The BBR test evaluates the deformation characteristics of the modified asphalt binder at low temperatures.

The asphalt binder that does not cause brittle fracture at low temperatures should be selected. The test is to evaluate the deflection characteristics that occur at this time by applying a load of 100 g (980 mN) for 240 s. We can evaluate the amount of deformation in 60 s and use this to determine creep stiffness, as shown in the following equation. The slope of creep stiffness at 60 s is measured and is defined as the m-value. From the creep load of the log loading time, creep stiffness is determined at the time of loading for 60 s, and this refers to the value measured after loading for 2 h at the actual site. The second variable to be determined in this test is the stiffness slope (m-value) with a 60 s loading. The creep stiffness determined in the BBR test must be 300 kPa or less, and the slope of the m-value must be at least 0.3 to satisfy the KS F 2390.

$$S(t)=\frac{{\mathrm{PL}}^{3}}{{4\mathrm{bh}}^{3}\Delta t}$$

S(t) = creep stiffness at time, t = 60 s;
P = applied constant load, 980 mN;
L = distance between beam supports, 120 mm;
B = beam width, 12.5 mm;
H = beam thickness, 6.25 mm;
Δt = deflection at time, t = 60 s.

Table 9 shows the results of the BBR test for the modified asphalt binder. Items that do not satisfy the specification of creep stiffness of 300 kPa or less and slope (m-value) of 0.3 or more are displayed in Table 9. The test results at −6 °C met the standard values of the specimen test results for all PCB contents of AP-3 and AP-5 asphalt binders. The specimen using 20% PCB did not satisfy the standard value at −12 °C. In the case of −18 °C, all test results were beyond the standard value.

## 3. Conclusions

The research presented in this paper aimed to evaluate the improvements in the performance of a modified asphalt binder with pyrolysis carbon black. Conventional laboratory tests and Superpave PG Binder tests were conducted. Despite the possible limitations of the number of tests, the following conclusions can be drawn:

(1) The specific gravity of PCB in this study was 1.000 g/cm^3^, and the specific surface area of PCB was 58 m^2^/g. The measured heavy metal concentration of PCB was 850~1239 for Ti and 86~490 for Pb.
(2) The average penetration values (recorded five times) of AP-3 (PG 58-22) and AP-5 (PG 64-22) were 116 and 91.7. As the amount of pyrolytic carbon black used increased, the penetration value of the asphalt binder tended to decrease.
(3) The rotational viscosity value tended to increase with pyrolysis carbon black, and the rotational viscosity value decreased with the testing temperature. The experiment results were measured to 3.0 Pa-s or less at the specification of 135 °C.
(4) As the pyrolysis carbon black content of the original asphalt binder increased, the value of G*/sinδ also increased, and all experimental values passed the reference value of 1.00 kPa or more. In the case of the short-term aged asphalt binder, the G*/sinδ value also increased as the pyrolysis carbon black content increased.
(5) The test results at −6 °C met the standard values of the specimen test results for all PCB contents of AP-3 and AP-5 asphalt binders. The specimen using 20% PCB did not satisfy the standard value at −12 °C. In the case of −18 °C, all test results were shown to exceed the standard value.

## Figures and Tables

**Table 1:** The amount (unit: ton) of waste tires generated by year.

Year	2018	2019	2020	2021
Tire replacement	297,785	281,876	280,457	292,629
Scrap car	84,312	102,287	95,895	91,171
Sum	364,297	384,163	376,352	383,800

**Table 2:** The major characteristics of pyrolysis carbon black.

Type	Adsorption (mg/g)	DBP (mL/g)	BET (m^2^/g)	Ash (wt. %)	Particle Size	Major Components				
						C	H	N	O	H_2_O
N220	121	114	119	0.1	345	98.2	0.2	-	1.1	0.4
N330	82	102	883	0.5	375	97.8	0.2	-	1.0	0.4
N660	36	90	35	0.2	425	98.4	0.4	-	03	0.7
PCB	54	71	58	11.3	355	90.9	0.37	0.23	3.53	2.05

**Table 3:** Penetration test for the modified asphalt binder (unit: 0.1 mm).

PCB %	0%		5%		10%		15%		20%	
	Avg.	Std.	Avg.	Std.	Avg.	Std.	Avg.	Std.	Avg.	Std.
AP-3	116.2	1.72	102.8	1.60	98.6	1.50	94.4	1.36	84.2	1.47
AP-5	91.6	1.02	82.8	1.17	78.6	1.02	74.2	1.33	67.6	1.36

**Table 4:** Softening point (unit: °C).

PCB %	0%		5%		10%		15%		20%	
	Avg.	Std.	Avg.	Std.	Avg.	Std.	Avg.	Std.	Avg.	Std.
AP-3	43.4	0.49	44.8	0.75	43.4	0.49	43.4	0.49	44.2	0.40
AP-5	44.2	0.40	44.4	0.49	45.0	0.89	45.2	075	47.4	0.49

**Table 5:** Measured shear modulus and phase angle for short-term aged asphalt binder (AP-3).

AC	Original Asphalt Binder						Short-Term Aged Asphalt Binder					
Temp.	52 °C		58 °C		64 °C		52 °C		58 °C		64 °C	
PCB %	G*	δ	G*	δ	G*	δ	G*	δ	G*	δ	G*	δ
0%	5.09	85.36	2.20	86.53	1.01	87.35	11.38	82.31	4.74	84.59	2.61	86.40
5%	5.64	85.98	2.40	87.39	1.09	88.52	11.28	82.81	486	84.91	2.19	86.61
10%	6.41	86.18	2.74	87.54	1.24	88.65	12.76	83.14	5.38	85.18	2.39	86.79
15%	7.45	86.27	3.20	87.62	1.44	88.71	11.03	83.51	4.60	85.53	2.08	87.12
20%	8.21	84.69	3.50	87.70	1.60	88.59	16.00	83.90	6.80	85.80	3.00	87.30

**Table 6:** Measured shear modulus and phase angle for short-term aged asphalt binder (AP-5).

AC	Original Asphalt Binder						Short-Term Aged Asphalt Binder					
Temp.	52 °C		58 °C		64 °C		52 °C		58 °C		64 °C	
PCB %	G*	δ	G*	δ	G*	δ	G*	δ	G*	δ	G*	δ
0%	6.22	85.25	2.69	86.74	1.20	87.96	13.29	81.99	5.40	84.30	2.31	86.05
5%	7.08	85.63	2.96	87.03	1.30	8825	15.25	82.17	6.25	84.43	2.66	86.25
10%	7.30	85.63	3.18	87.09	146	88.33	15.71	81.68	6.51	84.11	2.88	86.03
15%	8.50	85.91	3.66	87.29	1.63	88.46	20.41	82.47	8.42	84.66	3.65	86.40
20%	9.04	86.28	3.85	87.62	1.78	88.68	20.44	83.63	8.32	85.54	3.65	87.07

**Table 7:** Measured shear modulus and phase angle for long-term aged asphalt binder.

AC	AP-3						AP-5					
Temp.	22 °C		25 °C		28 °C		22 °C		25 °C		28 °C	
PCB%	G*	δ	G*	δ	G*	δ	G*	δ	G*	δ	G*	δ
0%	1861.0	49.3	1310.0	53.5	813.4	56.7	3557.0	47.1	2228.0	50.6	1384.0	54.0
5%	3031.0	50.4	1856.0	53.9	1141.0	57.1	4125.0	46.7	2582.0	50.4	1597.0	53.9
10%	4300.0	52.8	2813.0	55.8	1593.0	59.7	6307.0	48.5	3891.0	51.3	2427.0	54.7
15%	6294.0	51.7	3885.0	55.2	2347.0	58.6	5872.0	53.9	3483.0	57.9	2051.0	61.5
20%	4289.0	55.1	2582.0	58.7	15,030	62.2	5086.0	48.6	3182.0	52.3	1949.0	55.8

**Table 8:** G*sinδ of asphalt binder for fatigue cracking.

AC	AP-3			AP-5		
Temp.	22 °C	25 °C	28 °C	22 °C	25 °C	28 °C
0%	1411	1052	680	2603	1722	1119
5%	2335	1499	958	3003	1989	1290
10%	3423	2329	1376	4721	3037	1981
15%	3519	3189	2750	4724	2949	1802
20%	3519	2206	1329	3814	2515	1612

**Table 9:** BBR test results (creep stiffness and m-value) for AP-3.

AC	Original AP						RTFO AP					
Temp.	−6 °C		−12 °C		−18 °C		−6 °C		−12 °C		−18 °C	
PCB%	Stiffness	m	Stiffness	m	Stiffness	m	Stiffness	m	Stiffness	m	Stiffness	m
0%	84.3	0.38	158.2	0.38	332.1	0.31	84.2	0.38	160.4	0.35	332.4	0.30
5%	95.1	0.38	153.7	0.33	317.6	0.30	95.5	0.42	177.2	0.35	340.5	0.29
10%	7359	0.48	234.2	0.36	505.5	0.29	124.0	0.36	201.2	0.34	419.0	0.28
15%	72.7	0.48	228.6	0.33	565.3	0.28	135.5	0.36	255.8	0.30	417.5	0.28
20%	151.7	0.34	338.7	0.29	504.7	0.29	181.6	0.39	319.6	0.25	500.0	0.26
